# Associations Between Th17 Cell Markers (IL-23R, CCR6, and IL-17) and Clinical Profiles in Sjögren’s Disease

**DOI:** 10.3390/diagnostics15222909

**Published:** 2025-11-17

**Authors:** Erika Fabiola López-Villalobos, Jose Antonio Garcia-Espinoza, Mariel García-Chagollán, Jefte Felipe Uribe-Martínez, Sergio Cerpa-Cruz, José Francisco Muñoz-Valle, Claudia Azucena Palafox-Sánchez, Edith Oregon-Romero

**Affiliations:** 1 Doctorado en Ciencias Biomédicas, Centro Universitario de Ciencias de la Salud, Universidad de Guadalajara, Guadalajara 44340, Jalisco, Mexico; erikafabiola.lopez@academicos.udg.mx; 2 Laboratorio de Análisis Clínicos e Investigación Traslacional, Centro Universitario de Ciencias Exactas e Ingenierías, Universidad de Guadalajara, Guadalajara 44430, Jalisco, Mexico; 3 Departamento de Inmunología, Instituto de Investigaciones Biomédicas, Universidad Nacional Autónoma de México, Coyoacán 04510, Ciudad de México, Mexico; antoniojafar25helios@gmail.com; 4 Facultad de Ciencias de la Salud, Universidad Anáhuac México, Campus Norte, Huixquilucan 52786, Estado de México, Mexico; 5 Instituto de Investigación en Ciencias Biomédicas (IICB), Centro Universitario de Ciencias de la Salud, Universidad de Guadalajara, Guadalajara 44430, Jalisco, Mexico; chagollan@academicos.udg.mx (M.G.-C.); drjosefranciscomv@cucs.udg.mx (J.F.M.-V.); claudia.palafox@academicos.udg.mx (C.A.P.-S.); 6 Servicio de Reumatología, Hospital Civil “Fray Antonio Alcalde”, Guadalajara 44200, Jalisco, Mexico; jefteuribemtz@gmail.com (J.F.U.-M.); sergio.cerpa@academicos.udg.mx (S.C.-C.)

**Keywords:** Sjögren’s disease, Th17 cell heterogeneity, IL-23R^+^ T cells, immunophenotyping, flow cytometry, cytokine profiling, lipid metabolism

## Abstract

**Background/objectives:** Sjögren’s disease (SjD) is an autoimmune disorder characterized by lymphocytic infiltration and inflammation leading to exocrine gland dysfunction. Th17 cells play a central role in autoimmune pathology and are defined by markers such as IL-23R, CCR6, and IL-17. However, the combined characterization of these markers and their relevance in SjD remain poorly understood. **Methods:** Forty-one participants were enrolled, including twenty-two patients with SjD and nineteen control subjects (CS). Peripheral blood immunophenotyping was performed using multicolor flow cytometry, and serum cytokine concentrations were quantified within a multiplex assay. Non-parametric analyses were conducted using the Mann–Whitney U test and Spearman’s rank correlation. **Results:** Compared with CS, patients with SjD exhibited higher frequencies of CD3^+^CD4^+^IL-23R^+^ T cells and elevated IL-23 levels. The proportion of CCR6^+^IL-23R^+^ T helper cells tended to be higher in SjD than in controls, although this difference did not reach statistical significance (8.8% vs. 5.3%, *p* = 0.056). Within clinical subgroups, anti-Ro-negative patients showed increased frequencies of CD3^+^CD4^+^IL-23R^+^ cells. Patients with hypertriglyceridemia displayed reduced frequencies of CCR6^+^IL-23R^+^IFN-γ^+^ cells, whereas normal HDL levels were associated with CCR6 expression and IL-17A production. **Conclusions:** These findings highlight the heterogeneity of Th17 cells in Sjögren’s disease and reinforce the involvement of the IL-23/IL-23R axis in disease pathogenesis. Exploratory associations between Th17 subsets and lipid parameters suggest a potential immunometabolic interplay that warrants further investigation. Together, these data provide a more comprehensive view of Th17 dynamics in SjD and establish a foundation for future mechanistic studies in larger cohorts and tissue-specific contexts.

## 1. Introduction

Sjögren’s disease (SjD) is a chronic autoimmune disorder characterized by lymphocytic infiltration of the exocrine glands, leading to tissue damage and destruction. Clinically, it primarily manifests with oral and ocular sicca symptoms and occurs more frequently in women during the fourth or fifth decades of life [1,2].

The immunopathogenesis of SjD is not fully understood; however, it has been proposed that the disease develops through autoimmune epithelitis associated with lymphocytic infiltration. Activation of salivary gland epithelial (SGE) cells may be triggered by genetic or environmental factors, including viral infections. SGE cells function as antigen-presenting cells by expressing costimulatory and MHC-II molecules and secreting cytokines and chemokines, thereby promoting the recruitment and activation of T and B lymphocytes as well as other innate immune cells. The subsequent activation of T and B cells leads to the production of autoantibodies (anti-Ro and anti-La), which perpetuate inflammation and tissue destruction [1,2].

A central player in the pathophysiology of SjD is the T helper (Th) cell, which orchestrates immune responses through cytokine secretion [3]. Th cells express the CD4 coreceptor and are subdivided into subsets such as Th1, Th2, Th17, and others, each defined by distinct receptors, signature cytokines, and master transcription factors [4].

Among these subsets, Th17 cells have been implicated in several autoimmune diseases; however, their role in SjD pathogenesis remains unclear. Proposed mechanisms include the promotion of antibody class switching, modulation of autoreactive antibody glycosylation, and contribution to the formation of ectopic germinal centers [5].

Th17 cells are characterized by the expression of CCR6, CD161 and IL-23R; the secretion of IL-17 cytokines (IL-17A and IL-17F); and the master transcription factor RORγt [4,5]. Although these markers are essential for identifying the Th17 subset, most studies examining T-cell subsets have focused only on cytokines or surface receptors. In 2017, Verstappen et al. reported elevated frequencies of circulating Th17 cells (CCR6^+^CCR4^+^) in SjD patients [6], whereas Blokland et al. found similar percentages of peripheral IL-17-producing CD4^+^ T cells between SjD patients and healthy controls [7]. Fei et al. observed increased IL-17 expression in salivary glands correlating with lesion severity, as well as significantly higher frequencies of CD4^+^IL-17^+^ cells in the peripheral blood of SjD patients [8]. Although these markers are relevant, they are not exclusive to the Th17 subset. Therefore, to address this limitation, a combined immunophenotypic approach incorporating multiple markers may enable a more precise identification of Th17 cells and their functional heterogeneity.

The IL-23 receptor (IL-23R) is a key marker of the Th17 subset. IL-23R signaling maintains, supports, and expands the Th17 phenotype. The interaction between IL-23R and its ligand IL-23 upregulates the transcription factor RORγt, leading to IL-17 production [4,9]. Therefore, this study aimed to immunophenotype Th17 cells by assessing IL-23R, CCR6, and IL-17 expression and to examine their relationships with clinical parameters and disease severity in Sjögren’s disease.

## 2. Materials and Methods

### 2.1. Subject Recruitment

A cross-sectional study was conducted including twenty-two patients with Sjögren’s disease, diagnosed according to the 2016 ACR/EULAR classification criteria and without other overlapping systemic autoimmune diseases (inclusion criteria). All eligible patients during the study period were consecutively recruited from the Rheumatology Service of Hospital Civil “Fray Antonio Alcalde” (Guadalajara, Jalisco, Mexico). Only those fulfilling the inclusion criteria and providing written informed consent were enrolled. The study period extended from August 2019 to January 2022.

Non-inclusion criteria were active infection, recent severe trauma, or any condition compromising the immune system. General exclusion criteria, applied to both patients and control subjects (CS), included samples with insufficient blood volume or low peripheral blood mononuclear cell (PBMC) counts.

Ninety-eight percent of the patients were positive for minor salivary gland biopsy, and the remaining 2% were positive for anti-Ro antibodies. Focus score, disease duration, EULAR Sjögren’s Syndrome Disease Activity Index (ESSDAI), and Sjögren’s Syndrome Disease Damage Index (SSDDI) were obtained from medical records.

Additionally, nineteen unrelated healthy control subjects (CS) aged over 18 years, matched by age (±3 years) and sex, and without autoimmune, infectious, or other diseases that could affect immune function, were recruited from the community through open invitation. The health status of CS was verified using a standardized questionnaire and clinical records.

All participants provided written informed consent. The study was approved by the Ethics Committee of Hospital Civil “Fray Antonio Alcalde”, Guadalajara, Mexico (approval no. 088/19, 13 June 2019).

### 2.2. Antibody Quantification

Serum samples from SjD patients and CS were obtained from peripheral blood using vacuum tubes with gel and clot activator (BD Vacutainer^®^ SST^TM^, Franklin Lakes, NJ, USA) and stored at –20 °C until analysis.

Anti-Ro/SSA (52 and 60 KDa) and anti-La/SSB autoantibody levels were quantified using commercial ELISA kits (Orgentec Diagnostica GmbH, Mainz, Germany; codes 508 and 509, respectively) following the manufacturer’s protocol. According to the kit instructions, values < 25 U/mL were considered negative.

Antinuclear antibody (ANA) pattern detection was performed using an indirect immunofluorescence kit (Biosystem; Barcelona, Spain; code 44108) according to the manufacturer’s instructions. Slides containing HEp-2 cells were incubated for 30 min at room temperature (RT) with diluted serum samples (1:80, 1:160, 1:320, 1:640, 1:1280) and controls. After two PBS washes, slides were incubated with goat anti-human IgG-FITC/Evans antibodies for 30 min in a humidified chamber at RT. Following three PBS washes, slides were mounted with medium. ANA patterns were determined using an Axio Imager A2 microscope (Zeiss, Oberkochen, Germany) at 40× magnification and analyzed with Zen 3.5 Blue Edition software (Carl Zeiss Microscopy, Jena, Germany).

### 2.3. Turbidimetry Assays

Immunoglobulin (Ig) G, IgA, C-reactive protein (CRP), and rheumatoid factor (RF) levels were quantified using a Biosystem A15 Biochemistry Analyzer (Biosystem; Barcelona, Spain) with turbidimetric assays and reagents from the same supplier (IgG: code 1381; IgA: code 13082; CRP-hs: code 13927; RF: code 13922), following the manufacturer’s protocols.

### 2.4. Blood Lipid Profile and ESR

The erythrocyte sedimentation rate (ESR) was determined by the Wintrobe method using peripheral blood collected in EDTA-containing tubes. The lipid profile was analyzed using the Biosystem A15 Biochemistry Analyzer (Biosystem; Barcelona, Spain) with enzymatic and/or colorimetric assays and reagents from the same supplier (total cholesterol: code 12505; high-density lipoprotein (HDL): code 12557; low-density lipoprotein (LDL): code 12585; triglycerides: code 12528). Very low-density lipoprotein (VLDL) cholesterol was calculated as total cholesterol divided by five, and the atherogenic index as total cholesterol divided by HDL.

### 2.5. Flow Cytometry

Peripheral blood mononuclear cells were isolated by density gradient centrifugation using Ficoll-Paque^TM^ PLUS (Sigma-Aldrich, St. Louis, MO, USA; 17-1440-02) and cryopreserved in a solution containing 10% DMSO (Sigma-Aldrich; St. Louis, MO, USA), 40% FBS (Sigma-Aldrich, St. Louis, MO, USA), and 50% RPMI medium (Gibco; Brooklyn, NY, USA) at −80 °C until analysis.

PBMCs were adjusted to 1 × 10^6^ cells/mL and incubated for 4 h at 37 °C with 1 μg/mL Brefeldin A (BioLegend, San Diego, CA, USA; cat. 420601) to inhibit intracellular protein transport.

Cell viability was evaluated microscopically by trypan blue exclusion before staining, ensuring > 90% viable cells per sample. Samples with lower viability were not processed.

Extracellular staining was performed with anti-CD3 APC/Cyanine7 (BioLegend, San Diego, CA, USA; cat. 300426), anti-CD4 Alexa Fluor 488 (BioLegend, San Diego, CA, USA; cat. 300518), and anti-IL-23R (R&D Systems, Minneapolis, MN, USA; cat: FAB14001A) for 30 min at RT. Cells were then fixed and permeabilized for 1 h at 4 °C using the BD Fix & Perm^TM^ kit (cat: 562574). Intracellular staining was performed with anti-RORγt PE (BD Biosciences, San Jose, CA, USA; cat: 563081), anti-IFN-γ Brilliant Violet 421 (BioLegend, San Diego, CA, USA; cat: 502532), and anti-IL-17A PE/Cy7 (BioLegend, San Diego, CA, USA; cat: 353406) for 1 h at 4 °C. After two washes, cells were resuspended in 500 μL cold PBS (1×).

Samples were acquired on an Attune NxT cytometer (Thermo Fisher Scientific; Waltham, MA, USA), collecting at least 30,000 lymphocyte events per sample, and analyzed using FlowJo v10.7 software (BD Biosciences; San Jose, CA, USA).

### 2.6. Multiplex Assay

Cytokine quantification was performed using the Bio-Plex Pro^TM^ Human Th17 Cytokine Panel 15-Plex kit (#171AA001M, Bio-Rad Laboratories; Hercules, CA, USA) according to the manufacturer’s instructions. Samples were analyzed on a Bio-Plex^®^ MAGPIX^TM^ Multiplex Reader (Bio-Rad Laboratories). For this study, IL-17A (limit of detection (LD) ≥ 0.03 pg/mL), IL-17F (LD ≥ 0.13 pg/mL, IL-23 (LD ≥ 2.7 pg/mL), and IFN-γ (LD ≥ 0.26 pg/mL) levels were reported.

### 2.7. Statistical Analysis

Normality was assessed using the Shapiro–Wilk test. As most variables did not follow a normal distribution, non-parametric tests were applied. Results are presented as medians with interquartile ranges (25th–75th percentile), as appropriate.

Group comparisons between Sjögren’s disease patients and control subjects were performed using the Mann–Whitney U test. Correlations between variables were analyzed with Spearman’s rank correlation.

Statistical significance was set at *p* < 0.05. Analyses were conducted using SPSS v24 (IBM; Armonk, NY, USA), GraphPad Prism v8 (GraphPad Software Inc.; San Diego, CA, USA), and Python v6.1.4 (Python Software Foundation; Wilmington, DE, USA).

## 3. Results

### 3.1. Clinical and Demographic Characteristics

The study included forty-one participants: twenty-two patients with Sjögren’s disease (SjD) and nineteen control subjects (CS). Only one participant was male; the remainder were female. The median age of the SjD group was 56 years, with a median disease duration of 3 years. The demographic and clinical characteristics of both groups are summarized in Table 1. No significant differences were observed in IgA concentrations or lipid profile parameters between the SjD and CS groups.

### 3.2. Immunophenotyping of Th17 Cells

**Figure 1:**
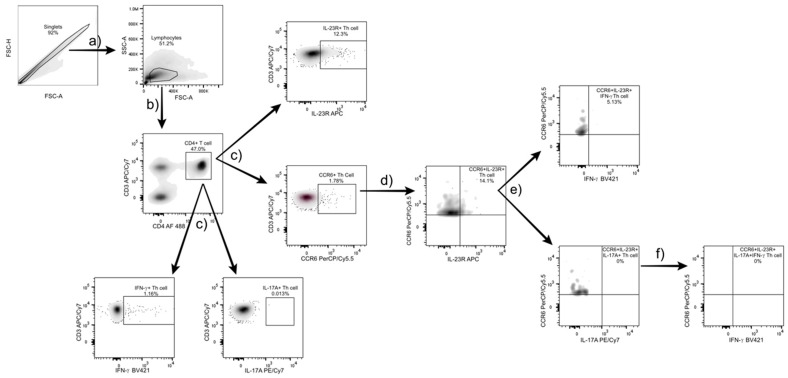
Gating strategy for Th17 and Th17.1 subset identification. (**a**) Lymphocytes were identified based on forward and side scatter (FSC/SSC) parameters from the singlet population, excluding doublets by FSC-A/FSC-H. (**b**) CD3^+^CD4^+^ T helper cells were gated from the lymphocyte population. (**c**) Within this population, specific markers (CCR6, IL-23R, IL-17A, and IFN-γ) were used for further gating. (**d**) From the CCR6^+^ population, a double gate with IL-23R was applied to identify CCR6^+^IL-23R^+^ Th17 cells. (**e**) Within this subset, IL-17A^+^ and IFN- γ^+^ populations were analyzed to distinguish IL-17A^+^ Th17 cells and CCR6^+^IL-23R^+^IFN-γ^+^ cells, respectively. (**f**) Finally, double-positive IL-17A^+^IFN-γ^+^ Th17.1 cells were identified. All gates were defined using fluorescence-minus-one controls, and data were analyzed with FlowJo v10.7 software (BD Biosciences, USA).

Th17 cells were characterized as follows. First, T helper cells were identified within the lymphocyte region using CD3 and CD4 markers. To define Th subsets, Th17-associated markers (CCR6, IL-23R, IL-17A) and IFN-γ (as a Th1 marker) were analyzed, and subgates were created for each marker. From the CCR6^+^ Th cell population, a double gate with IL-23R was applied to identify Th17 cells (Figure 1c). Within this population, further subgating with IL-17A and IFN-γ distinguished IL-17^+^ Th17 cells and Th17.1 cells (characterized by IFN-γ secretion). Finally, IL-17A^+^ Th17 cells co-expressing IFN-γ were gated to define the IL-17A^+^IFN-γ^+^ Th17.1 subset. The gating and analysis strategy used for identifying Th17 subsets is shown in Figure 1.

### 3.3. Th17 Population Frequencies

**Figure 2:**
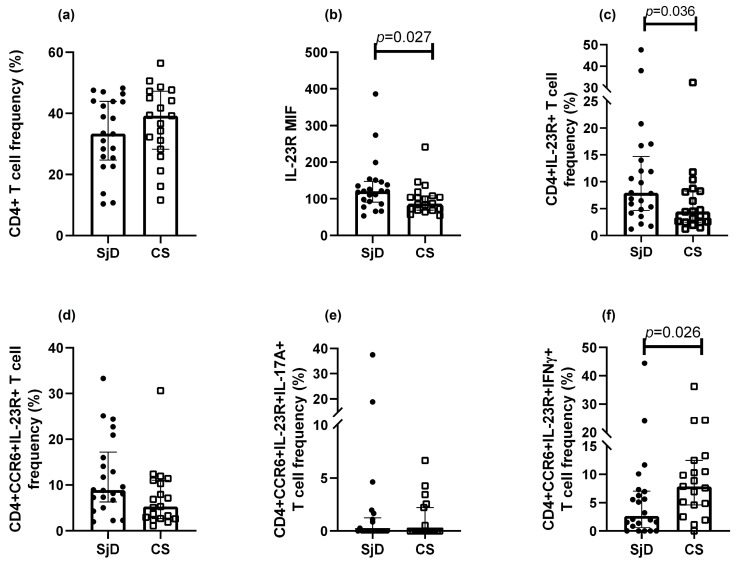
Th17 populations in SjD and CS. Analysis of Th17 subsets was performed in patients with SjD and CS. (**a**) “No differences were observed in CD4⁺ lymphocyte frequencies between SjD and CS groups. Significant differences were observed in IL-23R median intensity of fluorescence (MIF) and in the frequency of IL-23R^+^ Th lymphocytes in SjD compared with CS (**b**,**c**). CCR6^+^IL-23R^+^ T cells tended to be more frequent in SjD patients, although the difference did not reach statistical significance (**d**). Lower frequencies of CCR6⁺IL-23R⁺IL-17A⁺ cells were observed in both groups (**e**), whereas CCR6^+^IL-23R^+^IFN-γ^+^ cells were reduced in this group (**f**). Other marker combinations showed no significant differences. Data are presented as medians with interquartile ranges. MIF: median intensity fluorescence. Each point represents one subject (● = SjD; □ = CS).

As shown in Figure 2, there was a trend toward lower frequencies of CD4^+^ T cells in SjD compared with CS (33.4% vs. 39.2%, respectively) (Figure 2a). IL-23R^+^ Th cells were significantly increased in SjD relative to CS (7.9% vs. 4.4%; *p* = 0.036) (Figure 2b,c). A non-significant increase in CCR6^+^IL-23R^+^ Th cells was also observed in SjD (8.8%) compared with CS (5.3%) (*p* = 0.056) (Figure 2d).

In contrast, IL-17A^+^ Th cells were detected at low to undetectable levels. Moreover, when additional markers were included to define Th17 subsets, their frequencies progressively decreased. Interestingly, the Th17.1 population (CCR6^+^IL-23R^+^IFN-γ^+^ Th cells) was higher in CS than in SjD (Figure 2e). Meanwhile, the Th17 subset defined as CCR6^+^IL-23R^+^IL-17A^+^ Th cells approached 0% in both groups (Figure 2f). No significant differences were observed in other populations.

### 3.4. Cytokine Levels

**Figure 3:**
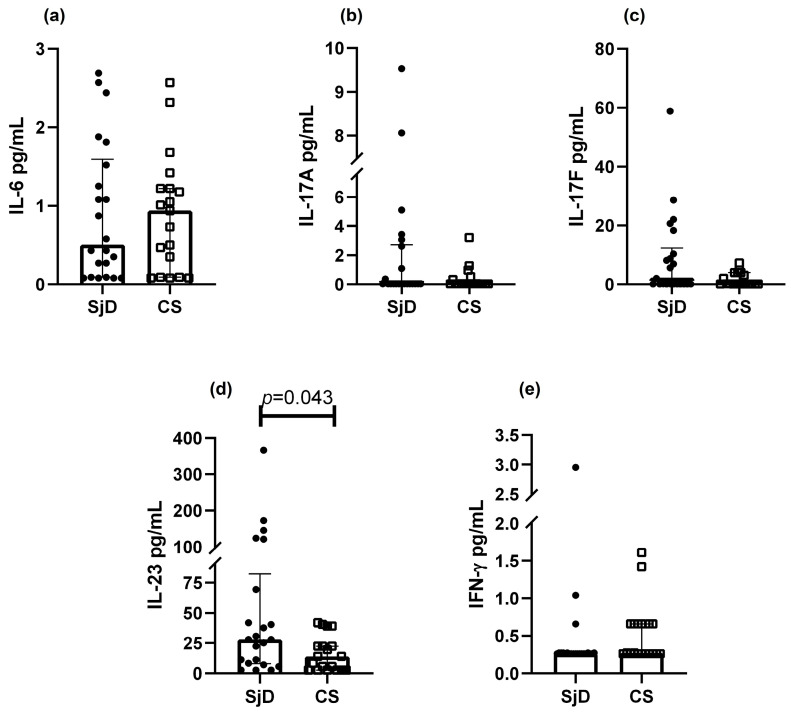
Th17 cytokine levels in SjD and CS. Analysis of Th17-related cytokines was performed in patients with SjD and CS. A non-significant trend toward lower IL-6 levels was observed in SjD (**a**), whereas IL-23 concentrations were significantly higher in this group (**d**). No significant differences were detected for IL-17A, IL-17F, or IFN-γ (**b**, **c** and **e**; respectively). Data are presented as medians with interquartile ranges. Each point represents one subject (● = SjD; □ = CS).

Cytokines associated with the Th17 phenotype were analyzed (Figure 3). IL-23 levels were significantly higher in SjD compared with CS (45.0 vs. 34.0 pg/mL, *p* = 0.043) (Figure 3d). A non-significant trend toward higher IL-6 levels was observed in CS (Figure 3a). Median concentrations of IL-17A, IL-17F, and IFN-γ did not differ between groups.

### 3.5. Comparison Between Th17 Populations and Clinical Parameters

Following immunophenotyping, Th17 subsets were analyzed in relation to clinical parameters (Table 2). Among antibody profiles, anti-Ro-negative patients exhibited a higher frequency of IL-23R^+^ cells (*p* = 0.043). Patients with hypertriglyceridemia (triglycerides > 150 mg/dL) showed a lower frequency of Th17.1 cells (CCR6^+^IL-23R^+^IFN-γ^+^) compared with those with normal triglyceride levels. In addition, patients with normal HDL concentrations (>40 mg/dL) showed associations with CCR6, IL-17, and their combinations with other markers, as summarized in Table 2.

Differences were also observed in lipid parameters across clinical subgroups of SjD. Patients positive for anti-La antibodies had higher total cholesterol (negative: 169.07 vs. positive: 221 mg/dL; *p* = 0.036) and LDL (negative: 54.77 vs. positive: 71.3 mg/dL; *p* = 0.036). Patients with elevated IgG levels displayed lower triglycerides (negative: 145.54 vs. positive: 103.37 mg/dL, *p* = 0.036) and lower VLDL (negative: 29.11 vs. positive: 20.67 mg/dL; *p* = 0.036). Conversely, RF-positive SjD patients presented with lower total cholesterol (negative: 224.38 vs. positive: 204.81 mg/dL; *p* = 0.043) and lower LDL (negative: 72.39 vs. positive: 50.14 mg/dL; *p* = 0.029).

### 3.6. Clinical Correlations

Spearman’s correlation analysis was performed to assess relationships between clinical parameters, cytokines, and Th17 populations. Among cytokines, IL-6 correlated positively with IL-23R expression (rho = 0.447, *p* = 0.037) and negatively with CCR6 MIF (rho = −0.464, *p* = 0.029). Interestingly, CD4^+^ T cells correlated negatively with IFN-γ (rho = −0.470, *p* = 0.027) and positively with IL-17A (rho = 0.452, *p* = 0.035). No other cytokine correlations reached statistical significance.

**Figure 4:**
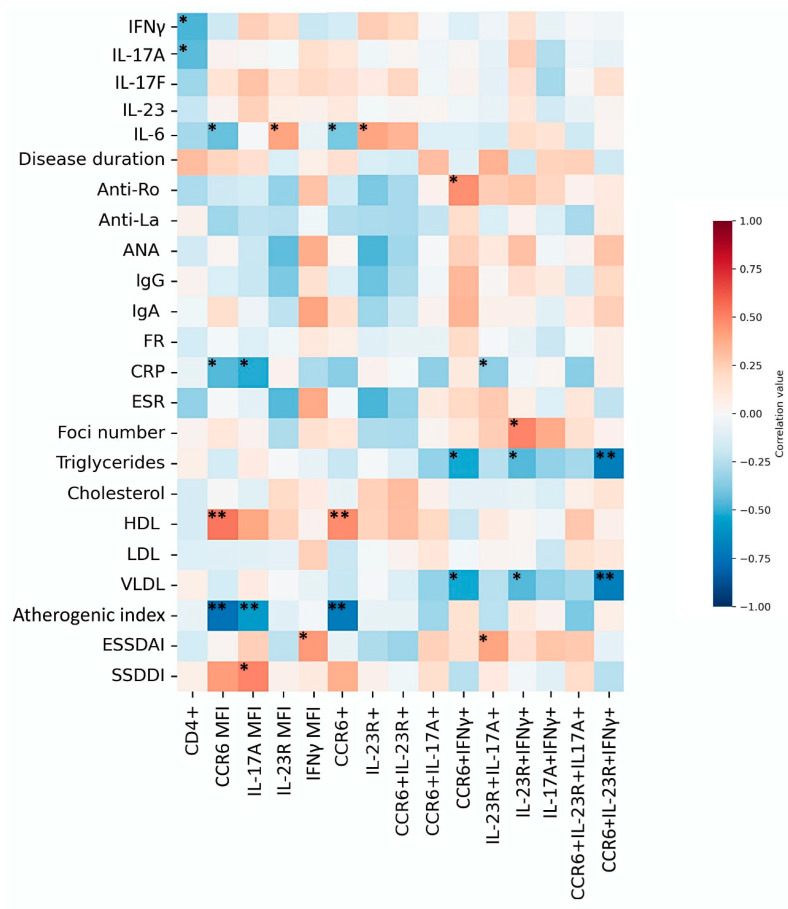
Heatmap of correlations between Th17 populations, cytokines, and clinical parameters. The heatmap depicts Spearman’s correlation coefficients between Th17 subsets, cytokine levels, and clinical parameters. Positive correlations are shown in red and negative correlations in blue. Statistical significance is indicated as *p* < 0.05 (*), *p* < 0.01 (**).

Several associations with clinical characteristics are summarized in Figure 3. Notably, strong positive correlations were found between HDL levels and both CCR6 MIF (rho = 0.618, *p* = 0.003) and CD4^+^CCR6^+^ cells (rho = 0.552, *p* = 0.009). Triglyceride levels correlated positively with Th17.1 cells (rho = 0.681, *p* = 0.001). In contrast, the atherogenic index correlated negatively with CCR6 MIF (rho = −0.848, *p* < 0.0001), CD4^+^CCR6^+^ T cells (rho = −0.805, *p* < 0.0001), and IL-17A MIF (rho = −0.552, *p* = 0.009). Additional associations are shown in Figure 4.

## 4. Discussion

The presence of specific markers—surface receptors (CCR6, IL-23R), the key cytokine IL-17A, and the master transcription factor RORγt—defines Th17 cells. Nevertheless, many studies rely on one or two markers to classify these cells, an approach that can be imprecise given their plasticity, particularly when cytokines are used as defining criteria [10]. For this reason, in the present study we evaluated Th17 cells using three markers to achieve a more accurate characterization and to explore their relationship with the clinical characteristics of patients with Sjögren’s disease (SjD).

Our results showed higher frequencies of CD3^+^CD4^+^IL-23R^+^ cells, increased IL-23R MIF, and a trend toward higher CCR6^+^IL-23R^+^ Th cells in SjD compared with CS. Conversely, IL-17A expression was very low in both groups. These findings are consistent with previous reports but also highlight ongoing controversy due to differences in marker selection. For example, Verstappen et al. observed elevated Th17 cells defined as CD45RA^−^CXCR5^−^CXCR3^−^CCR4^+^CCR6^+^ [6], whereas Aqrawi et al. reported no differences when defining Th17 cells as CXCR3^−^CCR6^+^ [11]. Li et al. found that IL-17A was mainly produced by CD4^+^CD161^+^ T cells [12], Alunno et al. described CD4^+^IL-17^+^ frequencies that did not differ between rituximab- and DMARD-treated patients [13], and Kang et al. observed higher CD4^+^IL-17A^+^ frequencies in SjD than in controls [14].

Th17 cells can display dichotomous behavior depending on marker expression and inflammatory potential. Pathogenic Th17 cells are often characterized by higher IL-23R expression and proinflammatory profiles. The IL-23/IL-23R axis plays a central role in maintaining Th17 cells, as IL-23 promotes RORγt activity, IL-17 production, and inhibition of IL-10, thereby sustaining inflammation [15,16].

By incorporating multiple markers, our study revealed that not all Th subsets fulfilled the complete panel of Th17-defining characteristics. Furthermore, the proportion of cells meeting these criteria decreased as additional markers were included, underscoring the heterogeneity within the Th17 population—a phenomenon also reported in rheumatoid arthritis [17].

The Th17 phenotype, defined as CCR6^+^IL-23R^+^IL-17A^+^, proved challenging to detect, primarily due to the low expression of IL-17A in peripheral blood. It is plausible that IL-17A^+^ cells are preferentially localized within salivary gland tissue [18,19,20]. Alternatively, CD4^−^CD8^−^ T cells may represent a main source of IL-17A rather than conventional CD4^+^ T cells [21].

Interestingly, the putative Th17.1 population (CCR6^+^IL-23R^+^ IFN-γ^+^) was less frequent in SjD than in CS. To our knowledge, no previous studies have analyzed this subset using the combined-marker approach applied here. One possible explanation is that this population is enriched at sites of local inflammation, whereas our analysis was limited to peripheral blood.

Several studies support tissue-specific distribution of Th17-related responses. Mieliauskaite et al. reported increased IL-23R and IL-17A expression in the salivary glands of SjD patients compared with non-autoimmune sicca controls [19]. Similarly, Nanke et al. identified IFN-γ^+^, IL-17^+^, and IFN-γ^+^IL-17^+^ cells within SjD salivary glands [22]. In murine models, disruption of the CCR6/CCL20 axis with neutralizing antibodies reduced Th17 infiltration in ocular tissues during dry eye disease [23]. Likewise, CD25-deficient mice with Sjögren-like keratoconjunctivitis sicca displayed increased CD4^+^ and CD8^+^ T cells in conjunctiva, along with elevated IL-17A and CCL20 mRNA expression in corneal and conjunctival tissues compared with wild-type mice [24]. Moreover, transfer of CD4^+^ T cells overexpressing RORγt into Rag2^−^/^−^ mice induced sialadenitis [25].

Nevertheless, the detection of Th17.1 cells in CS reflects the inherent plasticity of T cells and suggests that these populations do not necessarily exert pathogenic effects. Instead, their presence underscores the complexity of T-cell subsets and their potential roles in maintaining immune homeostasis under physiological conditions.

IL-23R expression has been strongly linked to Th17 plasticity, particularly the shift toward a Th1-like phenotype. Stimulation with IL-12 and IL-23, via STAT4 activation, promotes IFN-γ production and drives this transition [15]. Such plasticity may be relevant for mounting adequate immune responses and could be present at basal levels in healthy individuals [15,26]. However, further research is required to clarify this phenomenon.

Cytokine evaluation in our cohort revealed higher IL-23 levels in SjD patients, whereas IL-6, IL-17A, IL-17F, and IFN-γ levels did not differ between groups. Consistent with these findings, our group previously reported elevated IL-23 but no significant differences in IL-6 or IL-17A levels in SjD [27]. Although higher IL-23 concentrations may be associated with expansion of IL-23R^+^ Th cells, no direct correlations were observed. It remains possible that IL-23 contributes to maintaining the inflammatory state through induction of cytokines such as IL-22 and GM-CSF while simultaneously suppressing IL-10 [15].

Comparisons between clinical parameters and Th17 populations yielded additional findings. Anti-Ro-negative patients showed a higher frequency of IL-23R^+^ cells. This may relate to the presence of germinal centers (GCs) in the salivary glands of anti-Ro–positive patients, as our group has previously reported [28]. In such cases, Th17 cells may localize within GCs, acting as B-cell helpers by inducing isotype class switching [29,30], thus reducing their frequency in circulation. However, minor salivary gland biopsies were not analyzed here, which would be necessary to confirm this observation at the tissue level.

Unexpected observations emerged in the lipid profile analysis. Although our primary aim was not to explore lipid-Th17 relationships, we examined whether lipid levels differed between SjD and CS. As shown in Table 1, no significant group differences were found, in contrast to Zhang et al. [31]. Nonetheless, several associations were observed.

Patients with normal IgG levels exhibited higher triglyceride and VLDL concentrations (within non-pathological ranges). RF-negative patients showed higher total cholesterol (> 200 mg/mL) and LDL levels (within normal limits). Conversely, patients with hypertriglyceridemia had lower frequencies of Th17.1 cells, whereas those with normal HDL levels displayed associations with Th17 populations lacking IFN-γ. These findings suggest that HDL may influence CCR6 and IL-17 expression (supported by our correlation analyses), though additional studies are required to confirm this.

Lipid metabolism has been proposed as a regulator of Th17 polarization, with fatty acid synthesis (FAS), acetyl-CoA carboxylase, and the mTORC1 pathway driving Th17 marker expression and differentiation through epigenetic mechanisms. Activation of the mTORC1–FAS axis promotes glycolysis and de novo lipid synthesis, enhancing RORγt activity and IL-17 production, whereas its inhibition favors a regulatory phenotype [3,30,32]. This may partially explain why systemic lipid availability—such as variations in HDL, VLDL, or triglycerides—could indirectly modulate Th17 differentiation through metabolic cues.

HDL participates in lipid transport to the liver [33]. Although it may not directly determine Th17 differentiation, it could promote a metabolic environment that supports lipid synthesis and activation of the mTORC1/FAS axis [32,34]. Moreover, HDL has been shown to inhibit inflammatory cytokine production, thereby reducing the inflammatory milieu and limiting immune cell activation [35,36].

In contrast, lower triglyceride levels could promote Th17.1 cell development through IFN-γ expression. Our data also revealed that VLDL levels negatively correlated with IFN-γ populations, a pattern consistent with triglycerides. Because VLDL transports triglycerides from the liver to peripheral tissues [33], lower VLDL and triglyceride levels could favor polarization toward more pathogenic Th17 cells by limiting lipid substrates [32]. These observations suggest that maintaining lipid homeostasis is important. Furthermore, Th17.1 cells themselves may influence lipid metabolism through IFN-γ, which has been implicated in regulating triglyceride levels, lipolysis, lipogenesis, and fatty acid oxidation [37].

Although previous studies have described the influence of lipid metabolism on Th17 differentiation, to our knowledge no prior reports have directly examined how specific lipid parameters (triglycerides, HDL, and VLDL) modulate Th17 subsets in SjD. Therefore, this discussion is exploratory and provides a preliminary framework for understanding potential interactions between systemic lipid metabolism and Th17 polarization, meriting further mechanistic investigation.

This study has several limitations. Inclusion of RORγt as a marker, although essential for defining the Th17 phenotype, may have reduced the observed population due to decreased sensitivity when combining multiple markers. Although lipid profiling was informative, incorporating additional biomarkers such as apolipoproteins or lipoprotein subfractions could offer deeper insights. Moreover, by focusing exclusively on peripheral blood and not on local tissues (e.g., salivary glands), potentially relevant microenvironment-specific populations may have been overlooked. Finally, while the sample size was adequate for an exploratory study, a larger cohort would increase statistical power and improve generalizability.

## 5. Conclusions

Our findings highlight the heterogeneity of Th17 cells in Sjögren’s disease and reinforce the involvement of the IL-23/IL-23R axis in disease pathogenesis. We also observed exploratory associations between Th17 subsets and lipid parameters, suggesting a possible immunometabolic interplay that warrants further study. Collectively, these results advance the understanding of Th17 dynamics in Sjögren’s disease and provide a basis for future mechanistic investigations. Larger cohorts and tissue-specific analyses will be essential to confirm and expand these observations.

## Figures and Tables

**Table 1:** Demographic and clinical characteristics of participants.

Variables	SjD Patients (n = 22)	Control Subjects (n = 19)
**Demographics**		
Gender; Male/Female	1/21	1/18
Age, years; median (p25–p75)	56 (49.5–66.2)	55 (52–63)
Disease duration, years; median (p25–p75)	3.0 (2.0–6.5)	-
**Inflammatory markers**		
ESR, mm/H; median (p25–p75)	33.0 (21.2–42.7)	24.0 (13.0–33.0)
CRP, mg/L; median (p25–p75)	11.7 (8.9–16.1)	8.5 (2.4–12.8)
**Autoantibodies**		
RF, U/mL; median (p25–p75)	35.7 (8.3–89.3)	1.32 (0.0–4.77)
Anti-Ro, U/mL; median (p25–p75); %	2.2 (0.7–481.3); 41.7%	0.37 (0.15–0.91)
Anti-La, U/mL; median (p25–p75); %	2.9 (0.04–96.8); 29.2%	0.40 (0.82–0.94)
ANA, titer; median (range)	1:320 (1:160–1:1280)	-
**Clinical parameters**		
IgG, mg/dL; median (p25–p75)	1563.1 (1351.2–2358.6)	1338.4 (1060.3–1557.1)
IgA, mg/dL; median (p25–p75)	325.0 (261.7–449.5)	279.0 (250.0–321.0)
Total cholesterol, mg/dL; median (p25–p75)	211.5 (173.3–233.3)	195.7 (180.1–233.0)
HDL, mg/dL; median (p25–p75)	48.4 (31.1–60.3)	48.4 (42.8–58.8)
LDL, mg/dL; median (p25–p75)	67.8 (47.29–80.5)	62.6 (50.1–78.7)
VLDL, mg/dL; median (p25–p75)	22.0 (19.4–37.4)	27.62 (19.2–35.0)
Triglycerides, mg/dL; median (p25–p75)	110.4 (97.1–187.2)	143.1 (111.5–182.2)
Atherogenic index; median (p25–p75)	4.3 (3.6–5.3)	4.3 (3.5–5.1)
Foci number ≥ 1 focus/4 mm^2^; median (p25–p75)	2.0 (1.0–3.0)	-
SSDDI, score; median (p25–p75)	2.0 (1.0–3.0)	-
ESSDAI, score; median (p25–p75)	4.0 (2.0–5.7)	-
**Treatment**		
Prednisone, %	16.7	-
Hydroxychloroquine, %	83.3	-
Methotrexate, %	20.8	-
Azathioprine, %	37.5	-
Mycophenolate mofetil, %	8.3	-
Leflunomide, %	4.2	-
Rituximab, %	8.3	-
Abatacept, %	4.2	-
Cyclophosphamide, %	4.2	-

Data are shown as median (25th–75th percentile), median (range), or percentage unless otherwise indicated. Treatments represent the percentage of SjD patients receiving each medication. Abbreviations: ESR: erythrocyte sedimentation rate; CRP: C-reactive protein; RF: rheumatoid factor; ANA: antinuclear antibodies; IgG/IgA: immunoglobulin G/A; HDL: high-density lipoprotein; LDL: low-density lipoprotein; VLDL: very low-density lipoprotein; SSDDI: Sjögren’s Syndrome Disease Damage Index; ESSDAI: EULAR Sjögren’s Syndrome Activity Index.

**Table 2:** Th17 populations and clinical parameters groups in patients with SjD.

	Anti-Ro Positivity		
	Anti-Ro positive group (n = 9)	Anti-Ro negative group (n = 13)	*p*-value
IL-23R, MIF; median (p25–p75)	111.0 (7.75–121.5)	138.0 (94.95–176.50)	0.051
IL-23R^+^ Th cells %, median (p25–p75)	6.44 (2.94–8.90)	12.0 (5.59–18.90)	0.043 *
CCR6^+^IFN-γ^+^ Th cells %; median (p25–p75)	6.090 (2.13–7.58)	1.62 (1.06–3.40)	0.082
	**Anti-La positivity**		
	Anti-La positive group (n = 6)	Anti-La negative group (n = 15)	*p*-value
Cholesterol, mg/dL; median (p25–p75)	169.07 (136.28–212.38)	221.0 (204.81–236.83)	0.036 *
LDL, mg/dL; median (p25–p75)	54.77 (41.34–63.38)	73.45 (58.03–92.30)	0.036 *
	**Hypergammaglobulinemia positivity**		
	High IgG levels group (n = 11)	Normal IgG levels group (n = 9)	*p*-value
CCR6^+^IFN-γ^+^ Th cells %; median (p25–p75)	4.02 (2.64–6.76)	1.60 (1.06–6.12)	0.057
Triglycerides, mg/dL; median (p25–p75)	103.37 (87.11–118.18)	155.39 (109.97–216.79)	0.036 *
VLDL, mg/dL; median (p25–p75)	20.67 (17.42–23.64)	31.08 (21.99–43.36)	0.036 *
	**Rheumatoid factor positivity**		
	RF positive group (n = 11)	RF negative group (n = 10)	*p*-value
Cholesterol, mg/dL; median (p25–p75)	204.81 (137.95–223.27)	224.38 (209.80–242.18)	0.043
LDL; median (p25–p75)	50.14 (39.54–70.77)	75.82 (66.64–84.94)	0.029 *
	**Hypertriglyceridemia**		
	High Triglyceride levels group (n = 6)	Normal Triglyceride levels group (n = 15)	*p*-value
CCR6^+^IL-23R^+^IFN-γ^+^ Th cells %; median (p25–p75)	0.42 (0.0–2.58)	5.13 (1.61–7.32)	0.029 *
	**Abnormal HDL levels**		
	Low HDL levels group (n = 6)	Normal HDL levels group (n = 15)	*p*-value
CCR6, MIF; median (p25–p75)	99.10 (92.17–176.50)	286.00 (189.0–363.0)	0.003 *
IL-17A, MIF; median (p25–p75)	78.50 (54.55–103.42)	105.00 (94.3–118.0)	0.029 *
CCR6^+^ Th cells %; median (p25–p75)	6.51 (5.55–14.57)	28.40 (16.9–40.10)	0.018 *
IL-17A^+^ Th cells %; median (p25–p75)	0.00 (0.00–0.03)	0.05 (0.00–0.27)	0.018 *
IFN-γ^+^ Th cells %; median (p25–p75)	1.48 (0.82–2.29)	2.22 (1.19–5.45)	0.080
CCR6^+^IL-17A^+^ Th cells %; median (p25–p75)	0.00 (0.00–0.01)	0.14 (0.00–0.42)	0.036 *
CCR6^+^IL-23R^+^IL-17A^+^ Th cells %; median (p25–p75)	0.00 (0.00–0.00)	0.28 (0.00–1.98)	0.066

Comparisons were performed between Th17 subsets and clinical or laboratory parameters, stratified by antibody status (anti-Ro, anti-La, RF), hypergammaglobulinemia, and lipid profile (hypertriglyceridemia, HDL levels). Data are presented as medians (25th–75th percentile). Abbreviations: MIF: median intensity fluorescence; HDL: high-density lipoprotein; LDL: low-density lipoprotein; VLDL: very low-density lipoprotein; RF: rheumatoid factor. * *p* < 0.05.

## Data Availability

The original contributions presented in this study are included in the article. Further inquiries can be directed to the corresponding author.

## Abbreviations

The following abbreviations are used in this manuscript:

ANA	Antinuclear antibodies
CRP	C-reactive protein
CS	Control subjects
dL	Deciliter
ESR	Erythrocyte sedimentation rate
ESSDAI	EULAR Sjögren’s Syndrome Disease Activity Index
FAS	Fatty acid synthesis
GC	Germinal center
h	Hour
HDL	High-density lipoprotein
Ig	Immunoglobulin
IL-23R	Interleukin-23 receptor
L	Liter
LD	Limit of detection
LDL	Low-density lipoprotein
mg	Milligram
MIF	Median intensity fluorescence
mL	Milliliter
mm	Millimeter
PBMCs	Peripheral blood mononuclear cells
RF	Rheumatoid factor
RT	Room temperature
SGE	Salivary gland epithelium
SjD	Sjögren’s disease
SSDDI	Sjögren’s Syndrome Disease Damage Index
Th	T helper cell
U	Unit
VLDL	Very low-density lipoprotein
